# Inclusive Leadership and Taking-Charge Behavior: Roles of Psychological Safety and Thriving at Work

**DOI:** 10.3389/fpsyg.2020.00062

**Published:** 2020-02-20

**Authors:** Hao Zeng, Lijing Zhao, Yixuan Zhao

**Affiliations:** ^1^School of Economics and Management, Jiangxi Science and Technology Normal University, Nanchang, China; ^2^Business School, Nanjing University, Nanjing, China

**Keywords:** inclusive leadership, taking-charge behavior, psychological safety, thriving at work, self-determination theory, social information processing theory

## Abstract

Taking charge is an important form of proactive behavior that sustains organizational survival and individual development. Learning how to motivate employees to engage in taking-charge behavior has become one of the hot topics in the field of organizational management. Despite considerable research investigating the factors influencing taking charge, less attention has been paid to leadership-related factors, such as inclusive leadership. Based on the self-determination theory and the social information processing theory, we examined the mediating roles of psychological safety and thriving at work in the relationship between inclusive leadership and taking-charge behavior. We collected data in two stages from 205 pairs of employees and their supervisors at 17 companies in mainland China. Specifically, the results revealed that inclusive leadership promoted employees’ taking-charge behavior first through psychological safety and then through thriving at work. The results describe a more detailed mechanism underlying the formation of taking-charge behavior. The results further deepen our understanding of the path from inclusive leadership to employee behavior. These findings have theoretical implications for the taking-charge literature and managerial implications for practitioners.

## Introduction

In a dynamic, complex, vague, and uncertain environment, many organizations are decentralizing and beginning to redefine employee work roles. In an increasingly dynamic and changing context, companies not only require employees to efficiently perform tasks within their roles but also expect them to respond to challenges and engage in more proactive behaviors to build the core competitiveness of the organization. Taking charge is the constructive effort of employees to initiate self-improvement, improve organizational operations, and promote functional changes in the organization (Morrison and Phelps, 1999). Parker and Collins (2010) note that employee proactive behavior includes taking-charge behavior, speaking up, problem prevention, and personal innovation. Chiaburu et al. (2013) argue that taking-charge behavior is a change-oriented, organizational citizenship behavior that aims to identify and implement changes in work processes, products, and services. Studies have shown that taking-charge behaviors based on workflow or method improvement can enhance organizational adaptability and long-term viability (Moon et al., 2008; Parker and Collins, 2010). Employees’ taking-charge behavior not only enables better performance evaluation, job satisfaction, and affective commitment (Kim et al., 2015; Kim and Liu, 2017) but also fosters leadership potential and builds social networks (Fuller and Marler, 2009). In the Western context, taking-charge behavior can lead to positive individual and organizational results. However, Chinese employees are more willing to maintain harmony. Taking-charge behavior risks challenging the *status quo*; this behavior is in conflict with Confucian culture, which emphasizes “ren qing” (personal obligation), “face,” and “guan xi.” The following old Chinese saying is related to inclusiveness: “The sea can hold the water from 1000s of rivers; it’s big because of its capacity.” Therefore, the generation mechanism of taking-change behavior should be discussed in depth in the Chinese context.

By reviewing the literature, we found that previous studies mainly examined the factors influencing taking-charge behaviors from two aspects. The first aspect involves individual-level factors, such as self-efficacy (Moon et al., 2008), responsibility (Morrison and Phelps, 1999), psychological collectivism (Love and Dustin, 2014), psychological privilege (Klimchak et al., 2016), and prosocial motivation. For example, Mcallister et al. (2007) found that role sense and role effectiveness can significantly enhance employees’ willingness to engage in take-charge behaviors. The second aspect involves contextual factors, such as organizational support, social support (Backman et al., 2018; Feng et al., 2018), leadership support, working conditions (Bakr et al., 2019), distributional fairness, procedural fairness, and organizational development practice perception (Moon et al., 2008; Escribano and Espejo, 2010; Dysvik et al., 2016). For example, on the one hand, high-quality team-member exchanges (LMXs) (Kim et al., 2015) and support from leaders or colleagues are crucial factors driving employees to engage in taking-charge behavior (Love and Dustin, 2014; Backman et al., 2018; Feng et al., 2018). Social support and favorable work conditions could enhance the level of perceived job security and protect employees against psychological distress and job strain (Backman et al., 2018; Bakr et al., 2019). On the other hand, occupational stress in terms of low social support is related to general health (Finstad et al., 2019). Furthermore, the leadership-related factors, such as empowered leadership and transformational leadership (Li et al., 2015; Li J. et al., 2016), had been mentioned. Generally, there is a lack of research concerning the factors influencing taking-charge behavior from the leadership level. In the Chinese context, we aim to discuss whether and the extent to which taking-change behavior depends on the support and understanding of leaders.

Despite the mounting empirical evidence showing the direct and indirect effects of leadership on proactive behavior, there are still some gaps in our knowledge regarding the relationship between inclusive leadership and taking-charge behavior. First, although we already know that leadership has an impact on taking-charge behaviors, we also aim to investigate whether inclusive leaders motivate employees to engage in more proactive behaviors to enact change. Differing from empowered leadership and transformational leadership, inclusive leadership not only encourages employees to work independently and participate in decision-making but also recognizes their contributions, respects them, supports their growth, and embraces their failures. Second, the mechanism by which leadership impacts employee taking-charge behavior is far from fully revealed. As previously mentioned, social support (i.e., leadership support) can improve physical and mental health (Backman et al., 2018; Bakr et al., 2019). Inclusive leaders’ support, fair treatment, and fault-tolerant mechanisms can provide resources for employees and effectively predict the perception of psychological safety (Hirak et al., 2012). Taking-charge behavior challenges the *status quo*, which may cause conflicts and damage interpersonal relationships. Chinese people advocate harmony. We speculate that in the Chinese context, psychological safety is an important mediator between inclusive leadership and taking charge. Studies have shown that individuals with a sense of psychological safety are more likely to voice concerns or participate in creative work (Carmeli et al., 2010; Bienefeld and Grote, 2014). Third, in addition to psychological factors, the black box of the relationship between inclusive leadership and taking charge needs to be further clarified. Competence and motivation are the two most important factors influencing proactive behavior (Parker et al., 2010). Thriving at work is a state of mind resulting in feelings of vitality and learning by individuals. Learning implies improving abilities and self-confidence through the acquisition of knowledge and skills, while vitality represents a feeling of energy and enthusiasm (Porath et al., 2012). Niessen et al. (2017) also noted that the vitality and learning involved in thriving at work increase the willingness and ability of employees to engage in taking-charge behavior. Thus, our paper speculates that thriving at work may serve as a bridge between inclusive leadership and taking-charge behavior. However, the relationship between inclusive leadership and psychological safety, thriving at work, and taking-charge behaviors is seldom explored in existing research.

To address these research gaps, this research is based on the self-determination theory (SDT) and social information processing theory and aims to explore the mechanism by which inclusive leadership influences employees’ taking-charge behavior while considering the role of psychological safety and thriving at work. To ensure that the sample has a certain scope and representativeness, using a structured questionnaire, we collected data from employees and their supervisors at 17 companies in China. The proposed serial mediation model was tested through an analysis of two-wave surveys. To reveal causality among the variables and avoid common method bias, we utilize supervisor–subordinate dyad data.

### Development of Hypotheses

#### Inclusive Leadership and Taking-Charge Behavior

In the field of organizational behavior, Nembhard and Edmondson (2006) first proposed the concept of inclusive leadership. Scholars have defined inclusive leadership from three perspectives. First, the leader–member relationship perspective posits that inclusive leadership encourages employees to work independently and participate in decision-making. The leaders respect the employees, recognize the value of the employees, understand the employees’ needs, and provide support and advice (Hantula, 2009; Nishii and Mayer, 2009; Carmeli et al., 2010). Second, from the perspective of fairness, the key to inclusive leadership is to treat employees equally in diverse contexts (Nembhard and Edmondson, 2006). Third, the cultural background perspective holds that employees should be inclusive of different values and behaviors and tolerant of failures (Tang et al., 2015). Thus, inclusive leadership is a supportive, interactive, fair, fault-tolerant leadership style and an important organizational context variable that has a significant impact on its subordinate behaviors (Chen et al., 2002; Gong et al., 2009; Carmeli et al., 2013).

According to social information processing theory, the social environment contains various types of information that affect individual attitudes and behaviors. Individuals adopt appropriate behaviors through cognitive processing and their interpretation of social situations. To a large extent, environmental factors determine employees’ attitudes and behaviors (Salancik and Pfeffer, 1978). In the workplace, leaders represent an important source of social information, and employees focus on leaders and seek clues from them (Boekhorst, 2015). Compared to other leadership styles, inclusive leaders build a supportive atmosphere of equality, tolerance, and respect in the organization. On the one hand, inclusive leaders support their employees’ development, provide advice and assistance to their employees, and continuously enhance their employees’ ability to take charge and adapt to the environment. This leadership style provides information that the organization recognizes the employees’ values, promotes their growth, and encourages them to contribute; on the other hand, inclusive leaders allow different opinions, tolerate employee failures, and encourage innovation, which reduces the psychological and material costs of making an error. These aspects increase the willingness of employees to engage in taking-charge behavior. In short, employees interpret the organizational situation according to the inclusive leadership style, which has a strong prediction effect on taking-charge behavior. Therefore, hypothesis 1 is as follows:

*Hypothesis 1*. *Inclusive leadership will be positively related to taking-charge behavior in a work setting.*

#### Mediating Role of Psychological Safety

Psychological safety is the perception of interpersonal risk in the workplace (Edmondson, 1999, 2003). Psychological safety is a subjective perception of ease and security. When individuals feel safe in a work setting, they do not worry about the negative influences caused by self-expression, challenging their boss, or interpersonal conflict (Kahn, 1990); such employees are more likely to voice or take the initiative to change. In contrast, employees tend to remain silent or engage in evasive, passive behavior to protect themselves.

As previously mentioned, leadership style is an important contextual factor. When leaders’ behaviors exhibit more openness, accessibility, and availability (Carmeli et al., 2010), employees’ psychological safety is significantly improved. According to social information processing theory, the characteristics of inclusive leadership become the social information that is transmitted to the individuals in the organization. Other members of the organization will consider it reasonable and accept such information and follow suit. Thus, on the one hand, an organizational atmosphere of equality, tolerance, and trust helps enhance employees’ psychological safety and promotes individual learning. When organizations face change or innovation, employees will eliminate concerns regarding innovation failure, tend to propose new ideas, use new knowledge, and adopt new methods. Employees are more likely to seek their leaders’ help to avoid mistakes, improve their abilities, and build confidence through learning (Spreitzer and Porath, 2014). On the other hand, leaders’ attitude toward advice, respect, and trust can enhance employees’ psychological safety, promote positive emotions, and increase vitality at work. Psychological safety can help employees overcome the anxiety of learning. Therefore, this study believes that inclusive leadership has a positive impact on individuals’ psychological safety, which, in turn, has a positive impact on thriving at work.

*Hypothesis 2*. *Psychological safety plays a mediating role in the relationship between inclusive leadership and taking-charge behavior in a work setting.*

#### Inclusive Leadership and Thriving at Work

The socially embedded model of thriving at work (Spreitzer et al., 2005) notes that the social structural characteristics of work situations and job resources work together to promote thriving individuals. Based on SDT, inclusive leadership is an important organizational context that encourages employees to make decisions and creates an atmosphere of trust, respect, and recognition to meet employees’ autonomy, relatedness, and competence, which helps promote thriving at work. First, inclusive leadership encourages employees to work independently and participate in decision-making and creates a respectful and supportive atmosphere that satisfies employees’ autonomy. Inclusive leadership forms a powerful force for thriving. Second, the interactive and fair atmosphere helps create an equal, reciprocal, and pleasing environment. The members of the organization tend to establish a type of positive and friendly interpersonal relationship, meet the needs of employees’ relations, and enable individuals to experience learning and vitality. Once again, a fault-tolerant working style facilitates the exchange and sharing of information in an organization to meet the needs of competent employees. The collision of views stimulates innovation, promotes additional thriving, and marks individuals’ growth and progress.

Furthermore, the job resources (such as knowledge, emotions, and relationship resources) provided by an inclusive leader help individuals thrive at work. Conservation of resource theory (COR) argues that individuals strive to acquire, retain, and preserve crucial resources (Hobfoll, 2002). Individual job resources represent another important antecedent of individual thriving. Inclusive leadership helps employees achieve work goals, reduces job requirements, guards against physical and psychological depletion, and promotes individual growth and development (Demerouti et al., 2001). First, leaders are willing to listen to new ideas, encourage new ways of sharing new experiences, and help employees access knowledge resources. Second, a high level of leader–member exchange quality (LMX quality), a fair organizational atmosphere, and timely consultation and feedback are ways for employees to identify with the organization. Members obtain emotional and relationship resources, which, in turn, help individuals grow and develop. Therefore, based on the above analysis, hypothesis 3 is as follows:

*Hypothesis 3*. *Thriving at work plays a mediating role in the relationship between inclusive leadership and taking-charge behavior.*

#### Mediating Roles of Psychological Safety and Thriving at Work in the Relationship Between Inclusive Leadership and Taking-Charge Behavior

Based on hypothesis 1, hypothesis 2, and hypothesis 3, this study further assumes that inclusive leadership motivates employees to achieve thriving at work by improving the psychological safety of their subordinates in the work setting and ultimately adopting taking-charge behavior. Taking charge is an organizational citizenship behavior designed to improve organizational operations and promote organizational change (e.g., workflow, products, and services) (Morrison and Phelps, 1999; Chiaburu et al., 2013).

As mentioned above, according to social information processing theory, an inclusive leader provides clues for the cognitive and behavioral shaping of other members in the organization, laying the foundation for building an organizational climate. High-quality leader–member exchange equality, tolerance, and a respectful organizational atmosphere are conducive to employees showing themselves as much as possible to eliminate the negative effects of interpersonal conflicts, resulting in a more stable sense of psychological safety. Schein and Bennis (1965) also noted that psychological safety in the workplace is a necessary condition for individuals to participate in change. Second, psychological safety has a positive impact on thriving at work. Based on SDT, psychological safety can create a work situation that meets individuals’ competency, autonomy, and relatedness. Studies have shown that psychological safety has a positive impact on individual learning and vitality (Kark and Carmeli, 2009). When employees can make bold innovations without being blamed even if they make mistakes, their enthusiasm for learning will increase. When the organizational environment shows tolerance for mistakes and leaders provide counseling and friendship and develop trust with their employees, employees are more proactive at work and more likely to show determination and passion. Third, when employees have a higher level of thriving at work, they are more capable and motivated to make changes (Spreitzer et al., 2012). On the one hand, active learning and innovation motivate employees to take charge in relevant areas to resist risks; on the other hand, the vitality of work makes employees look forward to positive outcomes, and they expect to change their work content and methods to become more autonomous. Empirical studies indicate that thriving at work has a significant positive impact on employee performance, creativity, and innovation behavior (Porath et al., 2012; Jaiswal and Dhar, 2015; Wallace et al., 2016). Li M. et al. (2016) also confirmed that thriving at work is significantly positively correlated with employee change-oriented organizational citizenship behavior. Based on the above analysis, this study proposes hypothesis 4 as follows:

*Hypothesis 4*. *Psychological safety and thriving at work continuously mediate the relationship between inclusive leadership and taking-charge behavior.*

## Materials and Methods

### Participants

This study was conducted in 17 companies located in Jiangsu and Anhui provinces, China. The industries of these companies included trading, manufacturing, construction, etc. With the assistance of HR managers, we randomly selected and distributed the questionnaires to full-time employees and their direct supervisors. To reduce common method bias procedurally, we collected data in two phases with a 1-month interval between the phases. The purpose of phase 1 was to collect data related to the independent variable (i.e., inclusive leadership), mediators (i.e., psychological safety and thriving at work), and control variables, whereas the purpose of phase 2 was to acquire data related to the outcomes (i.e., taking-charge behavior).

Of the 320 employees contacted for data collection during phase 1, 263 employees returned their forms, yielding a response rate of 82.2%. Meanwhile, in phase 2, 263 employees were contacted for data collection, and 205 employees returned their forms, yielding a response rate of 77.9%. Of the respondents, 67.3% were male, and the average age was 33.56 years. Regarding the education level, 2.4% of the respondents had less than a primary school degree, 16.1% of the respondents had a high school or vocational school degree, 78.0% of the respondents had an undergraduate degree, and 3.4% of the respondents had a post-graduate degree. Finally, the average organizational tenure of the respondents was 6.79.

### Measures

According to the procedures recommended by Brislin (1986), we translated and back-translated the scales from English to Chinese to ensure that the original meaning was retained. Two proficient bilingual organizational behavior researchers conducted the translation. Furthermore, in previous studies, the validity of these scales has been verified in the Chinese context (Xu et al., 2019; Yan et al., 2019; Ye et al., 2019).

The items of each measure (taking-charge behavior, inclusive leadership, psychological safety, and thriving at work) were assessed on a seven-point Likert scale ranging from 1 = strongly disagree to 7 = strongly agree.

#### Taking Charge Behavior Scale (TCB; Parker and Collins, 2010)

We measured employee taking-charge behavior using the three-item TCB scale. The items include “How frequently does your subordinate try to improve procedures in his/her workplace?,” “How frequently does your subordinate try to propose new work methods that are more effective?,” and “How frequently does your subordinate try to implement solutions to pressing organizational problems?” In this study, Cronbach’s α of the TCB scale was 0.755.

#### Inclusive Leadership Scale (IL; Carmeli et al., 2010)

Inclusive leadership behavior was measured using the nine-item IL scale. Sample items include “The manager is open to hearing new ideas” and “The manager encourages me to access him/her regarding emerging issues.” In this study, Cronbach’s α of the IL scale was 0.891.

#### Psychological Safety Scale (PS; May et al., 2004)

We measured psychological safety using the three-item PS scale. Sample items include “I’m not afraid to be myself at work,” “I am afraid to express my opinions at work (r),” and “There is a threatening environment at work (r).” These items assessed whether the individuals felt comfortable to be themselves and express their opinions at work or whether there was a threatening environment at work. In this study, Cronbach’s α of the PS scale was 0.706.

#### Thriving at Work Scale (TW; Porath et al., 2012)

We used the 10-item TW scale to measure thriving at work, which included a learning latent factor and vitality latent factor. Sample items include “I find myself learning often” and “I feel alive and vital.” In this study, Cronbach’s α of the TW scale was 0.820.

#### Control Variables

We controlled for four employee demographic variables, including sex, age, level of education, and organizational tenure. Gender was a dummy variable (1 for men and 2 for women). Age was measured in number of years. Level of education was measured on a scale ranging from 1 (primary school or below) to 4 (graduate school). Organizational tenure was measured using the respondents’ self-reported years of working in the organization.

#### Analytic Strategy

First, we performed a descriptive analysis, reliability analysis, and correlation analysis using SPSS19. Second, confirmatory factor analyses (CFAs) were performed to examine the distinctive validity of our current variables, including taking-charge behavior, inclusive leadership, psychological safety, and thriving at work. Third, we estimated the path coefficients and three-path indirect effects along with the 95% bootstrapped confidence intervals (CIs) using the method recommended by Shrout and Bolger (2002) and Preacher and Hayes (2004). To date, bootstrapping is more advantageous than normal distribution–based significance tests (Shrout and Bolger, 2002).

## Results

Table 1 presents the means, standard deviations, correlations, and reliability estimates (Cronbach’s α) of all variables. All analyses were conducted with structural equation modeling (Mplus 5.21; Muthén and Muthén, 2007). Before forming the scales for the hypothesis testing, we assessed the construct validity of our measures using a CFA by comparing the measurement model with four competing models, which are described in detail in Table 2 (Anderson and Gerbing, 1988).

**TABLE 1:** Means, standard deviation, correlations, and reliability estimates of the study variables.

Variable	*M*	*SD*	1	2	3	4	5	6	7	8
(1) Age	33.56	7.913	–0.060							
(2) Tenure	6.789	7.432	0.038	0.736**	−0.263**					
(3) Inclusive leadership	5.176	0.741	0.026	0.012	0.020	0.030	**0.891**			
(4) Psychological safety	5.120	0.639	–0.028	0.008	0.090	0.091	0.437**	**0.706**		
(5) Thriving at work	5.270	0.609	0.036	–0.044	0.074	0.030	0.579**	0.469**	**0.820**	
(6) Taking-charge behavior	5.247	0.593	0.014	–0.001	–0.029	0.056	0.428**	0.400**	0.414**	**0**.**755**

*N* = 205; reliability coefficients are presented in bold. **p < 0.01.

**TABLE 2:** Fit indices of the alternative measurement models.

Measurement model	df	χ^2^	χ^2^/df	CFI	TLI	RMSEA
Single factor^a^	270	731.727	2.710	0.779	0.755	0.091
Two factors^b^	269	645.269	2.399	0.820	0.799	0.083
Three factors–1^c^	267	513.385	1.923	0.882	0.868	0.067
Three factors–2^d^	267	540.261	2.023	0.869	0.853	0.071
Four factors^e^	264	435.063	1.648	0.918	0.907	0.056

**N* = 205. CFI, comparative fit index; TLI, Tucker–Lewis index; RMSEA, root mean square error of approximation. ^*a*^All indicators load on a single factor. ^*b*^Inclusive leadership, psychological safety, and thriving at work load on one factor, and taking-charge behavior loads on one factor. ^*c*^Inclusive leadership and taking-charge behavior load on their respective factors, and psychological safety and thriving at work load on one factor. ^*d*^Inclusive leadership and psychological safety load on one factor, and thriving at work and taking-charge behavior load on their respective factors. ^*e*^Inclusive leadership, taking-charge behavior, psychological safety, and thriving at work load on their respective factors.*

As shown in Table 2, our four-factor measurement model was the best-fitting model and provided a reasonable fit for the data, supporting the unidimensionality of our measures as follows: comparative fit index = 0.918 and root mean square error of approximation (90% CI) = 0.056

**FIGURE 1:**
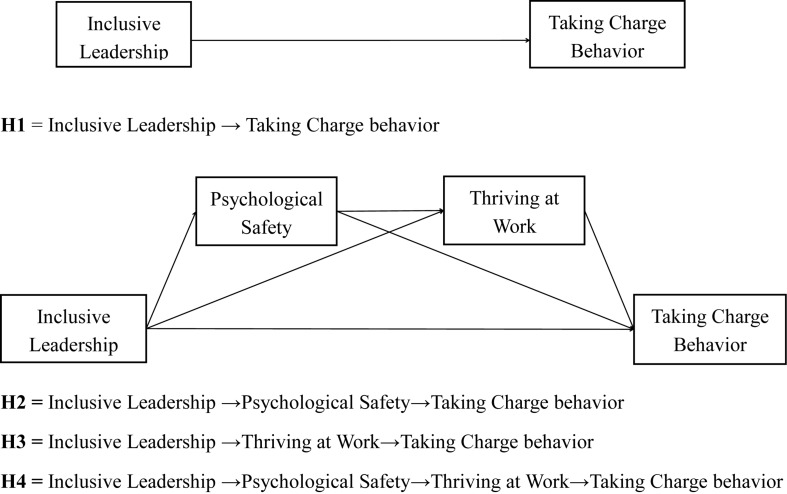
Empirical model.

As some measures (inclusive leadership, psychological safety, and thriving at work) are self-reported, we evaluated the impact of common method bias, which is highly problematic if a single latent factor accounts for the majority of the manifest variables’ variance. We tested for common method bias by loading each set of indicators on their latent variables and loading all items onto a fifth, common method latent variable. However, this five-factor model did not converge, which can be a widespread problem with a relatively small sample and a large number of items. Then, we conducted a Harman single-factor test (for a discussion, see Podsakoff et al., 2003) and found that the items did not significantly load onto a single factor. We concluded that common method bias was not a major concern in our analysis. In the structural model analysis, we used the Hayes macro PROCESS (Hayes, 2013) to estimate all path coefficients while simultaneously controlling for employee age, gender, education, and tenure. Table 3 shows the results. In our analytical model, we tested for a three-path mediated effect (Taylor et al., 2008). The advantage of this approach is that we were able to isolate the indirect effect of both mediators as follows: psychological safety (hypothesis 2) and thriving at work (hypothesis 3). This approach also allowed us to investigate the indirect effect passing through both mediators in a series (hypothesis 4; Taylor et al., 2008). Figure 1 illustrates these models. To test our mediation hypotheses, we used the analytical approach outlined by Shrout and Bolger (2002) and Preacher and Hayes (2004). This mediation approach directly tests the indirect effect between a predictor and the criterion variables through the mediator via a bootstrapping procedure (Efron and Tibshirani, 1993; Mooney and Duval, 1993), which addresses some weaknesses associated with the Sobel test (Shrout and Bolger, 2002; Preacher and Hayes, 2004). In Table 3, we provide the estimates of the indirect effects along with the 95% bias-corrected bootstrapped CIs of our path estimates.

**TABLE 3:** Path coefficients and indirect effects in the mediation models.

Direct effect	Estimate	*t*
IL→TC	0.183 (0.062)	2.953

**Indirect effect**	**Estimate**	**Bootstrap (bias-corrected bootstrap 95% confidence interval)**

IL→PS →TC	0.076 (0.028)	[0.031,0.142]
IL→TW→TC	0.067 (0.030)	[0.016,0.136]
IL→PS→TW→TC	0.016 (0.009)	[0.004,0.042]
Total effect	0.159 (0.047)	[0.080,0.268]

*Adapted from “Mediation and the Estimation of Indirect Effects in Political Communication Research,” by A. F. Hayes, K. J. Preacher, and T. A. Myers, in press; *Sourcebook for Political Communication Research: Methods, Measures, and Analytical Techniques*, by E. P. Bucy and R. L. Holbert, New York, NY, United States: Routledge. *N* = 205. Bootstrap confidence intervals were constructed using 5,000 resamples. Total effect (IL→TC) = 0.159 (0.047). Standard errors are shown in parentheses. IL, Inclusive Leadership Scale; TC, Taking Charge Behavior Scale; PS, Psychological Safety Scale; TW, Thriving at Work Scale.*

As predicted in hypothesis 1, inclusive leadership toward employees was positively related to taking-charge behavior. Hypothesis 2 stated that psychological safety mediates the path between inclusive leadership and taking-charge behavior. This hypothesis was supported. Hypothesis 3 was supported, as thriving at work mediates the path from inclusive leadership to taking-charge behavior. Hypothesis 4 stated that psychological safety and thriving at work sequentially mediate the relationship between inclusive leadership and taking-charge behavior. The analyses reported above show that psychological safety mediated the relationship between inclusive leadership and thriving at work and that thriving at work mediated the relationship between inclusive leadership and taking-charge behavior. We formally tested hypothesis 4 and found that inclusive leadership was associated with employees’ higher psychological safety and thriving at work, which was related to higher levels of taking-charge behavior.

## Discussion

Due to the uncertainty of the external environment and the limitation of organizational managers’ energy and capabilities, organizations expect to develop employees’ proactive behaviors to meet challenges. This study must identify ways to intrinsically inspire employees’ taking-charge behavior. The aim of this study was to investigate the effect of inclusive leadership, perceived psychological safety, and thriving at work on employees’ taking-charge behaviors in the Chinese context. The results support our hypotheses and reveal the following: (1) inclusive leadership is positively related to taking-charge behavior; (2) psychological safety is positively related to taking-charge behavior; (3) thriving at work is positively related to taking-charge behavior; and (4) psychological safety and thriving at work continuously mediate the relationship between inclusive leadership and taking-charge behavior.

### Theoretical Implications

The theoretical contributions of our study mainly include three aspects. First, from the perspective of leadership, the antecedents of taking-charge behavior were expanded. As mentioned earlier, the antecedents of taking-charge behavior are abundant, and studies have mainly focused on individual factors, organizational context factors, and leadership. From the perspective of leadership behavior, previous studies have examined the impact of transformational leadership, self-sacrificial leadership, empowered leadership, benevolent leadership, and abusive leadership on taking-charge behavior. Among these leadership styles, transformational leadership positively affects employees’ taking-charge behavior (Li J. et al., 2016; Homberg et al., 2017). Self-sacrificial leadership has a significant positive impact on employees’ taking-charge behavior. Furthermore, organizational identity plays a partial mediating role between these factors (Li R. et al., 2016). Other studies have shown that empowered and ethical leaders do not have a significant direct impact on employees’ taking-charge behavior (Lee, 2016; Qian et al., 2018). Xu et al. (2018) also examined the impact of benevolent leadership on employees’ proactive change behavior. In contrast, Ouyang et al. (2015) found that there is a negative relationship between abusive supervision and proactive behavior because the style of leadership reduces subordinates’ perceived insider status within an organization. In addition, Li et al. (2013, 2015) tested the relationship between team empowered leadership and employees’ taking-charge behavior. Furthermore, some studies have noted that deep supervisor–subordinate similarity perceived by employees has a significant impact on employee’s taking-charge behavior and that supervisors’ inclusiveness acts as a negative moderator in the relationship between supervisor–subordinate similarity and taking-charge behavior (Zheng et al., 2017). Studies have begun to focus on leaders’ inclusiveness and its impact on employees’ taking-charge behavior. Differing from other types of leadership, inclusiveness is a leadership style in which supervisors care about the needs of their subordinates, are good at listening to the opinions of their subordinates, and recognize their subordinates’ contributions. Our findings mainly reveal the positive impact of inclusive leadership on taking-charge behavior. Thus, in response to the call for more context models to reveal the relationship between leadership and taking-charge behavior (Xu et al., 2018), this study expanded existing research at the organizational level and provided an application context.

Second, based on SDT and social information processing theory, the variables mediating employees’ psychological safety and thriving at work were examined. Mediation in existing research is usually explained through traditional social psychology perspectives, such as the social exchange theory, social identity theory, social cognitive theory, motivation theory, etc. The mediators of the relationship between leadership and taking-charge behavior mainly include psychological cognitive factors (e.g., role-breadth, self-efficacy, entitlement, organizational identification, insider status, trust in leader, and identification with leader) (Li et al., 2013, 2015; Ouyang et al., 2015; Lee, 2016; Li J. et al., 2016), motivation (Homberg et al., 2017), emotion or attitude (e.g., positive affect and work engagement) (Fritz and Sonnentag, 2009; Xu et al., 2018), and individual behavior (e.g., feedback-seeking) (Qian et al., 2018). On the one hand, our research validates the positive impact of inclusive leadership on taking-charge behavior from the perspective of social information processing theory. Studies have shown that positive leadership can introduce a sense of job security and job satisfaction (Bakr et al., 2019), and the positive impact of inclusive leadership’s fault tolerance is highly important. On the other hand, thriving at work is introduced as a mediator based on SDT. Instead of examining mediators, such as cognitive and emotional variables, separately, thriving at work includes the two dimensions of learning and vitality, effectively integrating cognitive and emotional factors. Our research finds that inclusive leadership meets employees’ requirements (competence, autonomy, and relatedness) such that employees are more likely to thrive at work. Thus, this study comprehensively examines the mechanism by which inclusive leadership influences taking-charge behavior from both cognitive and emotional aspects and expands our knowledge regarding the role of leadership behavior in taking-charge behavior.

Third, this study builds a chained mediation model of inclusive leadership, psychological safety, thriving at work, and taking-charge behavior. Studies have noted that taking-charge behavior is a challenging, transformative, and risky proactive behavior (Mcallister et al., 2007; Parker et al., 2010). Therefore, whether employees take charge largely depends on the level of their psychological safety (Carmeli et al., 2010). Existing research shows that social support can protect employees against psychological distress (Feng et al., 2018), and inclusive leadership to subordinates can improve employees’ psychological safety level (Hirak et al., 2012) and stimulate employees’ state of learning (cognition) and vitality (emotion), which, in turn, positively affects their ability and willingness to engage in taking-charge behaviors. The results provide a more detailed mechanism underlying the formation of taking-charge behavior.

### Practical Implications

Our study advances the idea that it is important to practice inclusive leadership to enhance employees’ psychological safety, thriving at work, and taking-charge behaviors. First, from the perspective of the leader, the supervisor should be friendly, accessible, concerned with the needs of the subordinates, tolerant of different opinions, and tolerant of mistakes to a certain extent. Furthermore, leaders should provide support and job resources to their subordinates. For example, supervisory mentoring is beneficial to subordinates. The supervisor establishes a mentoring relationship with subordinates, provides career support and psychosocial support, and serves as a role model for subordinates (Scandura, 1992). Research has shown that among behaviors related to high-quality relationships, holding behaviors are effective job resources helping employees cope with changes and challenges (Ragins et al., 2016).

Second, from an organizational point of view, we should create an inclusive climate and consider a series of measures through work guidance and caring for employees to improve employees’ psychological safety to stimulate their willingness to take charge. Furthermore, training opportunities and supportive resources should be provided to enhance their capacities to take charge. For example, relevant policies encouraging learning and innovation should be formulated, new methods and new ways to solve problems should be adopted, and certain error indicators should be added to employees’ performance appraisal systems.

Finally, through special training, companies can enhance managers’ inclusive leadership qualities and capabilities. Organizations can first assess the leadership levels of existing management teams and find gaps in their inclusive leadership skills to determine the importance and difficulty of leadership training. In particular, an understanding of millennials’ values, viewpoints, requirements, and behavior patterns in the workplace could help supervisors build an inclusive mindset. For example, it is possible to establish a mentoring relationship between supervisors and subordinates to strengthen the frequency of communication, expand the scope of mutual learning, form benign interactions, and gain information.

### Limitations and Future Research Directions

Although our research has certain theoretical contributions and practical implications, there are still some research limitations. First, one potential disadvantage is related to the design strategy. Although we collected data at two time points, the relevant data are reported by supervisors and subordinates, which may lessen transient response biases and common method biases; however, the cross-sectional design still limits inferences of causality. Future studies should use longitudinal studies to confirm causality in theoretical models. Regarding the sample collection, we collected data from 17 Chinese companies, but whether the effect of inclusive leadership on taking-charge behavior can be generalized to other samples remains questionable. Future research should collect data more widely in various industries and countries to increase the generalizability of our findings. In addition, in this study, we did not control for other positive leadership styles (Wang et al., 2019), such as transformational leadership, ethical leadership, moral leadership, and benevolent leadership. Future research should control for the impact of similar leadership styles on taking-charge behavior to enhance the robustness of the results.

Second, this study expands the antecedents of taking-charge behavior but only examines the antecedents from the leadership perspective. As mentioned before, the antecedents of taking-charge behavior are extremely rich and mainly include individual factors (such as emotions, cognition, and personality traits) and organizational context factors (such as relationships, working conditions, organizational structure, and leadership). Taking-charge behavior is likely the result of the combined effect of individual factors and the organizational context. Existing research fails to integrate the various influencing factors, such as individuals and organizations, and thus ignores the interaction between individual factors and organizational factors. Future research may consider combining the causes of different types or levels of taking-charge behavior and comprehensively examine how multiple combinations promote or prohibit taking-charge behavior. For example, how can leadership be matched with the traits of employees to motivate employees to taking charge or render the implementation of leadership more effective?

Third, the mechanism by which leadership mediates taking-charge behavior needs to be further explored. Drawing upon SDT and social information processing theory, this study examines the impact of inclusive leadership on taking-charge behavior at both cognitive and psychological levels. Existing research is mainly based on the perspective of social exchange theory, social identity theory, social cognitive theory, and motivation theory, and future research could further expand the theoretical perspective of the relationship between leadership behavior and taking-charge behavior, such as by examining COR. According to this theory, resources are “individual characteristics, conditions, energy, etc. that make individuals feel valuable or a way to obtain them” (Hobfoll, 1989). Knowledge, skills, development opportunities, job autonomy, social relations, social support, and optimistic personality are all valuable resources for individuals. Inclusive leadership, as a positive leadership behavior, respects the needs of employees, affirms the value of employees, and tolerates the different views of employees. First, inclusive leadership provides positive psychological resources to help employees build a sense of psychological security and self-efficacy. Second, inclusiveness is an organizational support factor that increases job autonomy and is a valuable job resource (De Cuyper et al., 2012). Therefore, future research could expand from the perspective of resource gain and loss.

Fourth, future research should continue to explore the outcome variables of taking-charge behavior. In the existing literature, more research is concerned with the antecedents of taking-charge behavior, and exploration of the consequences is scarce. Future research could investigate the positive and negative effects of taking-charge behavior on individuals. For example, taking-charge behavior may have a negative impact on personal work–family balance (Greenhaus and Powell, 2006). Based on the integration model of proactive behavior (Bindl and Sharon, 2010), future research could also test the impact of taking-charge behaviors on outcome variables at the team level (e.g., team performance and team effectiveness) and organization level (e.g., organizational performance and innovation).

## Conclusion

The present study demonstrates how inclusive leadership motivates employees’ taking-charge behavior in the workplace, adapting to the dynamic environment. This study provides new insight into the relationship between inclusive leadership and taking-charge behavior and helps us better understand the impact of inclusive leadership on proactive behavior.

The contributions of this study concern different aspects. First, drawing upon SDT and social information processing theory, we establish a chained mediation theoretical model of inclusive leadership, employee psychological safety, thriving at work, and taking-charge behaviors. In China’s organizational context, we find that inclusive leadership has a positive effect on employees’ taking-charge behavior. Second, in addition to psychological factors, thriving at work lies in the integration of the cognitive and emotional aspects and plays a positive role in the relationship between inclusive leadership and taking-charge behavior. This study further deepens our understanding of the path between inclusive leadership and employee behavior. Third, the potential research value lies in encouraging more organizations to adopt an inclusive style of leadership in an era of change to stimulate taking-charge behaviors, improve organizational adaptability and innovation, and enhance employees’ satisfaction and personal growth (Kim and Liu, 2017).

## Data Availability Statement

All datasets generated for this study are included in the article/supplementary material.

## Ethics Statement

The studies involving human participants were reviewed and approved by the Ethics Committee (HREC) of the School of Economics and Management in Jiangxi Science and Technology Normal University. The patients/participants provided their written informed consent to participate in this study.

## Author Contributions

HZ contributed to the idea and wrote the full manuscript. LZ collected the data and run the data. YZ revised the full manuscript and proposed improvements.

## Conflict of Interest

The authors declare that the research was conducted in the absence of any commercial or financial relationships that could be construed as a potential conflict of interest.
